# Healthcare burden of rare diseases in Hong Kong – adopting ORPHAcodes in ICD-10 based healthcare administrative datasets

**DOI:** 10.1186/s13023-018-0892-5

**Published:** 2018-08-28

**Authors:** Annie Ting Gee Chiu, Claudia Ching Yan Chung, Wilfred Hing Sang Wong, So Lun Lee, Brian Hon Yin Chung

**Affiliations:** 1 Department of Paediatrics and Adolescent Medicine, Queen Mary Hospital, Hong Kong, SAR, People’s Republic of China; 2 Department of Paediatrics and Adolescent Medicine, The University of Hong Kong, Hong Kong, SAR, People’s Republic of China

**Keywords:** Rare diseases, ORPHAcodes, ICD-10, Disease burden, Prevalence, Inpatient cost, Hong Kong

## Abstract

**Background:**

The burden of rare diseases is important for healthcare planning but difficult to estimate. This has been facilitated by the development of ORPHAcodes, a comprehensive classification and coding system for rare diseases developed by the international consortium Orphanet, with cross-references to the 10th version of the International Classification of Diseases and Related Health Problems (ICD-10). A recent study in Western Australia made use of this cross-referencing to identify rare diseases-related admissions in health administrative datasets. Such methodology was adopted in Hong Kong, which has a population of 7 million comprising of 92% ethnic Chinese, with over 80% of admissions taking place in the public hospitals and available for review from the local public healthcare database.

**Main body:**

Our objective was to identify the inpatient healthcare burden of rare diseases in Hong Kong. We extracted admission records of all patients coded with one or more of the 1084 ICD-10 codes cross referenced with 467 ORPHAcodes during the study period from 1st January 2005 to 31st December 2016. We further analysed rare disease-related inpatient healthcare cost using a subset of patients admitted during 1st April 2015 – 31st March 2016. A total number of 546,673 admissions were identified, representing 3.2% of total admissions during the study period. By the end of the study, 109,535 patients were alive, representing 1.5% of the overall population. Prevalence of rare diseases was found to be 1 in 67 in the Hong Kong population. The most common rare disease category in the paediatric age group was ‘rare developmental defect during embryogenesis’; whereas that amongst adults was ‘rare haematologic disease’. The aforementioned subset of patients accounted for 330,091 inpatient-days, placing the estimated total inpatient cost for rare disease population at HKD$1,594,339,530 i.e. 4.3% of total inpatient cost in 2015–2016.

**Conclusion:**

Cross referencing between ICD-10 and ORPHAcodes may be adopted in different healthcare datasets for international comparison. Despite differences in the prevalence of individual disease, the disparity between rare disease prevalence (1.5%) and associated inpatient cost (4.3%) in Hong Kong reflects the importance of rare diseases in healthcare policies.

## Background

Rare diseases refer to conditions with rare occurrences in a population. They are often life threatening or chronically debilitating [1], but with limited treatment owing to lack of incentive in marketing (Rare Disease Act of 2002, United States). The number of rare diseases is estimated at 5000 to 8000. They are individually rare but collectively affect 6–8% of the European population [2] i.e. similar to the proportion of people living with asthma or diabetes [3].

The disease burden of rare diseases may be gauged either from the individual’s perspective or from its overall impact on the healthcare system. From the individual patient’s perspective, 25% of patients had to wait 5 to 30 years from disease onset to receiving a diagnosis; and in 40% the initial diagnosis was wrong, according to EURORDIS, a rare disease patient organization in Europe [4]. A similar study performed in Australia showed that 30% of the patients waited more than 5 years to receive a diagnosis, 60% saw more than 3 doctors before receiving a diagnosis and nearly half of the patients received at least one incorrect diagnosis during their diagnostic odyssey [5].

The impact of rare diseases on the healthcare system is harder to gauge. Firstly, there is a lack of universal definition for rare disease, with prevalence threshold varying across countries, ranging from 5 to 76/ 100,000 and an average threshold of 40/100,000 people [6]. Secondly, new diagnoses are fast emerging, with discovery of more than 100 new diseases per year from 2010 to 2015 [7]. As such there have only been a few studies on the healthcare burden of rare diseases, which mostly utilize national registries for rare diseases or national disability registry for estimation [8, 9]. They are helpful in delineating rare disease related demographics but rely on reporting from individual practitioners and cannot reflect more complicated information such as inpatient attendance or length of stay.

Currently a few rare disease nosologies exist, the most common ones being the Orphanet Rare Disease Ontology (ORDO) and Online Mendelian Inheritance in Man (OMIM). Whereas OMIM is mostly used in genetic databases, ORDO is a structured vocabulary for rare diseases derived from the orphanet database, which defines rare disease as occurring in less than 1 in 2000. It captures relationship between diseases, genes and other relevant features to form a useful resource for computational analysis of rare diseases, and has connections with other terminologies or classification system such as the 10th version of the International Classification of Diseases and Related Health Problems (ICD-10). Whereas ICD-10 contains only 500 unique codes for rare diseases, it is now overcome by the cross referencing with ORPHAcodes, a comprehensive classification and coding system for rare diseases developed by the international consortium Orphanet [10]. ORPHAcodes are now increasingly used by European healthcare systems for informatics tracing of rare diseases, and their introduction is fostered by National Action Plans and Strategies for Rare Diseases and recommended by the European Commission expert group on rare diseases.

A recent study by Walker et al. made use of health administrative data sets to identify admissions related to rare diseases in Western Australia [11]. This was achieved through cross referencing between ORPHAcodes and an Australian modification of ICD-10 (ICD-10-AM). Using back-and-forth translation, a panel of medical experts came up with 1084 disease codes and used this as the basis for calculation of healthcare burden relating to rare disease. This study was a paradigm shift that allowed for not only the calculation of prevalence but also that of healthcare cost relating to rare diseases [11]. It also provided an opportunity for international comparison using comparable methodologies.

Hong Kong has a population of 7 million with approximately 92% of the population being ethnic Chinese [12]. More than 80% of admissions take place in the public hospitals under the management of the Hospital Authority (HA) [13]. Within HA, all inpatient diagnosis are recorded using ICD-10, all of which are available in the Clinical Data Analysis and Reporting System (CDARS) in an unlisted and anonymous manner for research purposes.

Rare disease has recently gained more public awareness in Hong Kong, as evident by both the establishment of the Hong Kong Alliance of Rare Disease in 2014 and legislative council debates on rare disease in 2016–2017. The recent spotlight on rare diseases highlighted the lack of local epidemiological studies on rare diseases. To bridge this literature gap, we adopted the methodology described by Walker et al. [11] in the public healthcare administrative system of Hong Kong. In this study, we sought to i) estimate the prevalence and distribution of rare diseases in Hong Kong; ii) ascertain the number and proportion of admissions in rare diseases requiring inpatient care; and iii) estimate the inpatient-related healthcare cost in patients with rare diseases in Hong Kong.

## Methods

### Rare disease diagnostic coding

The Hospital Authority of Hong Kong adopts the Hong Kong Clinical Terminology Table (HKCTT) for diagnostic coding purposes. The HKCTT contains mapped codes to three versions of ICD-10, namely ICD-10 2001, ICD-10 2010, and ICD-10 Mental Health and Behavioural Disorders (ICD-10 MBD), which are searchable on the CDARS database [14] and reported to be reliable in previous epidemiological studies [15, 16].

We adopted the 1084 ICD-10-AM codes set out by Walker et al. [11] in our study, which were matched to 467 ORPHAcodes with back-and-forth translation and verified using period prevalence with exclusion of infectious diseases. Records corresponding to the above diagnostic coding were identified from the CDARS database.

### Study population

This retrospective study included all patients who had an inpatient admission at one or more of the hospitals under the HA with one or more of the above 1084 diagnostic codes between 1st January 2005 and 31st December 2016. Other extracted variables included the patients’ age at admission, gender, length of stay, and mortality (as defined by death recorded in the Hong Kong Death Registry).

### Data analysis

Demographic information about the study population and their admissions to public hospitals in Hong Kong, including the number of subjects, number of admissions, male to female ratio and age distribution were calculated.

Rare diseases were analysed by their ORPHAcode categories. All patients in the CDARS database were recorded in an unlisted and anonymous manner by a unique reference key (CDARS ID number, which is different from the Hong Kong Identity Card number). Multiple lines of entries with the same reference key were found when the same patient has been admitted into any of the hospitals under the HA system for more than once. The total number of admissions, total number of rare disease patients, and the number of patients in each disease category were calculated. Multiple admissions with one of the 1084 diagnostic codes for the same patient were included when considering the total number of admissions; whereas the reference keys were sorted and counted once only by their unique codes using Microsoft Excel when considering the total number of patients in the study and the number of patients in each disease category.

Patient’s age at admission was used for the paediatric and adult subgroup analysis, this could be calculated using the date of admission and the date of birth for each patient, which were recorded in the CDARS database. When considering the number of patients in the paediatric and adult population, it was presumed that some patients would have had admissions both before and after their 18th birthday, the age of the patient’s first admission was used because it was known that some rare diseases have an earlier onset than others.

The average, median, and interquartile range (25% and 75% quartile) of the length of stay in each disease category were also calculated. Instead of using the 95% confidence interval which assumed the data to follow a normal distribution, the interquartile range was used as the dataset was skewed by some patients with a very prolonged LOS.

The surviving number of patients on 31st December 2016 was identified for calculation of overall and category specific rare disease prevalence, using the Hong Kong Census data from the end of 2016 [12]. A subset of data between 1st April 2015 and 31st March 2016 (representing a fiscal year for the HA) was further identified. Using this set of data, the overall cost of inpatient stay for rare disease patients was calculated, which was assumed to be the same over the study period, with a unit cost of HKD$4830 (i.e. USD$619) per inpatient-day (acute and convalescent beds) according to the HA 2015–2016 data [13]. This cost was an actual mean cost calculated in the general population (including rare diseases patients) in 2015–2016. It was understood that the cost per inpatient-day would be highly variable among different diseases treated, however, disease-specific inpatient cost data is not available in the local setting.

Data analysis was performed using R (version 3.4.1).

## Results

### Whole cohort

A total of 144,444 patients were identified from the CDARS database, with male to female ratio of 1:1.05. There were 546,673 admissions, with the paediatric to adult admission ratio being 1:3.09 (see Table 1). The under 5 years age group had the largest total number of admissions, accounting for 17.8% of the total number of admissions in this cohort (see Table 2), and 7.1% of the under 5 years age group in the general population over the study period.

**Table 1:** Basic demographics of patients with rare diseases (12-year cohort from 2005 to 2016)

Study period	01–01-2005 to 31–12-2016
Total population	144,444
Total number of admissions	546,673
Male: female ratio	1:1.05
Paediatric (≤18 years): adult admission (> 18 years)	1:3.09

**Table 2:** Distribution of rare diseases related hospital admissions by age at admission in Hong Kong (2005–2016)

Age group	Number of admissions in the rare disease population (% of the total rare disease population)	Number of admissions in the general population (% of the total general population, including all diseases)
0–4	97,416 (17.8%)	1,373,714 (8.0%)
5–9	34,438 (6.3%)	259,111 (1.5%)
10–14	26,776 (4.9%)	208,713 (1.2%)
15–19	28,565(5.2%)	256,811 (1.5%)
20–24	26,808 (4.9%)	369,707 (2.2%)
25–29	27,598 (5.0%)	597,883 (3.5%)
30–34	28,001 (5.1%)	824,150 (4.8%)
35–39	21,554 (3.9%)	768,238 (4.5%)
40–44	17,983 (3.3%)	709,462 (4.2%)
45–49	21,336 (3.9%)	914,342 (5.3%)
50–54	28,092 (5.1%)	1,161,757 (6.8%)
55–59	30,965 (5.7%)	1,284,643 (7.5%)
60–64	30,686 (5.6%)	1,273,321 (7.5%)
65–69	28,174 (5.2%)	1,238,402 (7.2%)
70–74	25,249 (4.6%)	1,298,569 (7.6%)
75–79	27,861 (5.1%)	1,494,884 (8.7%)
80–84	23,493 (4.3%)	1,397,762 (8.2%)
85+	21,678 (4.0%)	1,659,092 (9.7%)
Total	546,673	17,090,561

The majority (95%) of the cohort had only one rare disease diagnosis pertaining to one rare disease category; 27,075 (5.0%) had rare disease diagnoses from two or more rare disease category. These patients were included in the calculations of all the respective disease categories but only counted once in the overall counts for patients and admissions. Over the study period, the rare disease category with the most patients was ‘rare developmental defect during embryogenesis’; whereas the rare disease category with the highest number of inpatient admissions (including repeated admissions) was ‘rare haematologic disease’. Within the paediatric age group, ‘rare developmental defect during embryogenesis’ was the most common rare disease category, whereas ‘rare haematologic disease’ was the most common rare disease category within the adult age group (see Table 3).

**Table 3:** Rare disease in Hong Kong at a glance (2005–2016)

Orphanet classification	Number of people (%)	Mean (median) age	Number of people <=18 years old (%)	Estimated prevalence, as of 2016
Rare developmental defect during embryogenesis disease	33,731 (21.9)	21.4 (11.9)	23,835 (70.7)	457 per 100,000
Rare haematologic disease	24,615 (16.0)	51.8 (37)	3331 (13.5)	334 per 100,000
Rare neurologic disease	23,707 (15.4)	56.1 (57.7)	4507 (19.0)	321 per 100,000
Rare neoplastic disease	14,556 (9.4)	70.6 (61.0)	757 (5.2)	197 per 100,000
Rare endocrine disease	9923 (6.4)	48.9 (53.7)	2893 (29.2)	135 per 100,000
Rare systemic or rheumatologic disease	8678 (5.6)	42.0 (48.5)	3411 (39.3)	118 per 100,000
Rare otorhinolaryngologic disease	7726 (5.0)	54.4 (60.0)	2052 (26.6)	105 per 100,000
Rare cardiac disease	6581 (4.3)	63.9 (65.0)	300 (4.6)	89 per 100,000
Rare respiratory disease	4810 (3.1)	21.4 (10.5)	3739 (77.7)	65 per 100,000
Rare inborn errors of metabolism	4287 (2.8)	57.1 (44.6)	707 (16.5)	58 per 100,000
Rare eye disease	3303 (2.1)	22.6 (9.9)	2422 (73.3)	45 per 100,000
Rare skin disease	2646 (1.7)	68.1 (79.8)	220 (8.3)	36 per 100,000
Rare renal disease	2532 (1.6)	12.9 (8.7)	2230 (88.1)	34 per 100,000
Rare hepatic disease	2455 (1.6)	69.8 (71.0)	66 (2.7)	33 per 100,000
Rare gastroenterologic disease	2447 (1.6)	72.0 (78.8)	24 (1.0)	33 per 100,000
Rare bone disease	1191 (0.8)	39.9 (37.0)	408 (34.3)	16 per 100,000
Rare gynecologic or obstetric disease	653 (0.4)	48.5 (46.0)	2 (0.3)	9 per 100,000
Rare urogenital disease	119 (0.1)	39.3 (34.1)	27 (22.7)	2 per 100,000
Rare immune disease	78 (0.1)	30.2 (35.7)	45 (57.7)	1 per 100,000
Rare odontologic disease	32 (0.0)	40.4 (45.2)	11 (34.4)	< 1 per 100,000
Rare abdominal surgical disease	5 (0.0)	56.1 (57.3)	0 (0.0)	1 per 1,000,000
Rare circulatory system disease	2 (0.0)	36.6 (36.6)	0 (0.0)	< 1 per 1,000,000
Total	144,444			

**Fig. 1:**
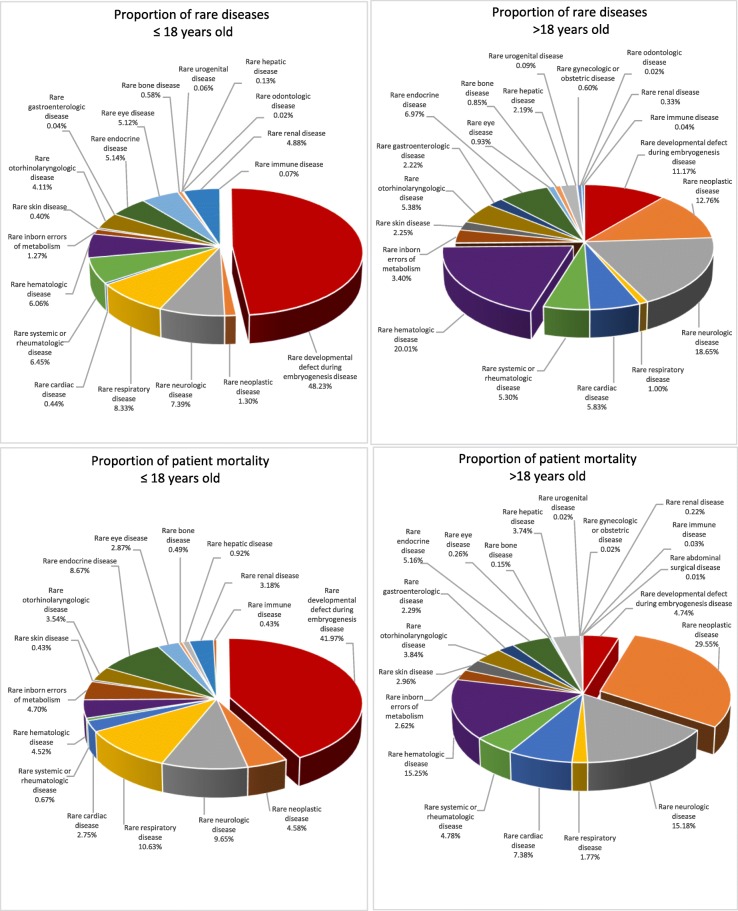
Proportion of rare diseases and mortality in paediatric and adult age patients from various rare disease categories

A total of 34,909 deaths were recorded over the study period (96.8% contributed by the adult population); 109,535 were alive (75.8%) as of 31 December 2016. Consequently, in December 2016 the whole rare disease population represented 1.5% of the Hong Kong population. Within the paediatric age group, ‘rare hepatic disease’ had the highest proportion of mortality (25.4%), whereas ‘rare neoplastic disease’ had the highest proportion of mortality within the adult age group (74.4%). The relative proportion of rare diseases and mortality of different rare disease categories in paediatric and adult age groups is represented in Fig. 1.

The average and median length of stay (LOS) were 6.7 days and 1 day respectively in the rare disease population. Within the under-five age group, the average and median LOS were 8.7 days and 1 day respectively.

‘Rare respiratory disease’ has the longest average length of stay, followed by ‘rare eye disease’. It should be noted that the median LOS are considerably shorter than that of the average LOS across all disease categories, suggesting that the dataset is skewed towards shorter LOS with some patients having very prolonged LOS (see Table 4).

**Table 4:** Discharges and LOS of inpatient healthcare amongst people with rare diseases in Hong Kong (2005–2016)

Cohort	Total admission over the study period	Total hospital stay in days	Mean (Median) LOS	25–75% quartile LOS
All discharges	546,673	3,635,917	6.7 (1)	1–4
Rare haematologic disease	135,503	418,635	3.1 (1)	1–149
Rare neoplastic disease	121,824	691,149	5.7 (1)	1–157
Rare developmental defect during embryogenesis disease	99,638	736,445	7.4 (1)	1–618
Rare neurologic disease	54,954	775,579	14.1 (3)	1–954
Rare systemic or rheumatologic disease	31,408	186,689	5.9 (1)	1–115
Rare endocrine disease	26,518	187,198	7.1 (1)	1–201
Rare cardiac disease	22,944	153,627	6.7 (3)	1–92
Rare inborn errors of metabolism	22,483	95,734	4.3 (1)	1–121
Rare otorhinolaryngologic disease	13,044	116,342	8.9 (2)	1–183
Rare respiratory disease	9345	284,592	30.5 (7)	1–290
Rare hepatic disease	6436	57,788	9.0 (4)	1–69
Rare gastroenterologic disease	5804	26,699	4.6 (2)	1–40
Rare eye disease	5709	141,814	24.8 (1)	1–195
Rare renal disease	5539	74,345	7.9 (2)	1–77
Rare skin disease	4811	67,255	14.0 (5)	1–95
Rare bone disease	2873	38,580	13.4 (2)	1–88
Rare immune disease	2219	5963	2.7 (1)	1–9
Rare gynecologic or obstetric disease	826	1156	1.4 (1)	1–2
Rare urogenital disease	259	713	2.8 (1)	1–2
Rare odontologic disease	33	46	1.4 (1)	NA^a^
Rare abdominal surgical disease	6	27	4.5 (3)	NA^a^
Rare circulatory system disease	4	26	6.5 (2)	NA^a^

*LOS* length of stay

^a^ NA due to limited observations for calculation

### 2015–2016 cohort

Further analysis was performed using the 1st April 2015 - 31st March 2016 cohort, which included 22,606 patients and 54,488 admissions. The mean length of stay was 6.1 days, which was marginally higher than that of the general population (5.8 days). The period prevalence of rare disease was estimated at 1 in 67 people using the Hong Kong Census data from the end of 2016 [12].

There were a total of 330,091 inpatient-days in the above period. Taking the unit cost per inpatient-day provided by the HA for the year 2015–2016, which was HKD$4830 (i.e. USD$619) per day, the estimated total inpatient cost was HKD$1,594,339,530 (i.e. USD$204,402,504), accounting for 4.3% of the total inpatient cost for all diseases in Hong Kong, in which the total inpatient cost for all diseases was estimated at HKD$37,467,184,230 (i.e. USD$4,803,485,158) (see Table 5).

**Table 5:** Total number of inpatients, admissions, inpatient-days and total inpatient costs of rare disease population (2015–2016)

	Total number of inpatients	Total number of admission	Total inpatient-days	Total inpatient cost (HKD)^a^
2015–2016 rare disease population (% as compared to the general population)	22,606 (2.9%)	54,488 (5.06%)	330,091	1,594,339,530 (4.26%)
General population (all diseases including rare diseases)	777,616	1,077,325	7,757,181	37,467,184,230

*HKD* Hong Kong dollars, *LOS* length of stay

^a^Unit cost per inpatient-day in general (acute and convalescent) beds (as of 2015–2016 actual price): HKD$4830

## Discussion

The current study analysed the inpatient burden of healthcare from patients with rare disease. The 467 studied rare diseases represented 1.5% of the Hong Kong population by 2016, and accounted for approximately 4.3% of the inpatient hospital costs for that year. This disparity does not only contribute to the immediate healthcare cost, but will also have substantial social and economic implications for patients, family members, and carers [17].

The utilisation of CDARS allowed identification of all patient data in Hong Kong under the HA, which handles more than 80% of all admission in Hong Kong. CDARS is proven to be a reliable source of health administrative data [18], and the adoption of the methodology by Walker et al. in CDARS allowed international comparison of rare disease related epidemiological data. Both studies showed that the inpatient cost of rare diseases is disproportionally higher than its prevalence. Likewise, rare developmental defect during embryogenesis, which accounted for nearly one-third of the list of ORPHAcodes, has the highest patient load in both this study and the Western Australia cohort [11].

There were however, some notable difference in disease distribution between the two cohorts. In the Western Australian cohort, rare neoplastic diseases had the highest adult prevalence, whereas in the current cohort, this was superseded by rare haematologic diseases. We postulate that such difference could be attributed to the difference in disease burden across ethnic groups, eg. higher prevalence of thalassaemia major, lower prevalence of rare neoplasms such as Hodgkin lymphoma in ethnic Chinese, which is the predominant in Hong Kong. We also note a difference in disease burden between the paediatric and adult population, the most significant one being the predominance of rare developmental defect during embryogenesis disease (48.2%) in the paediatric population, which only constitute 11.2% of rare diseases in the adult population. On the other hand, the proportion of patients with rare haematologic diseases increases from 6.1% in the paediatric rare disease population to 20% in adulthood. This increment is matched by rare neoplastic diseases (from 1.3% in paediatric age group to 12.8% in adulthood) and rare neurologic diseases (from 7% in paediatric age group to 18% in the adulthood). Of interest is that rare inborn errors of metabolism, which is traditionally thought to predominate in childhood, is actually more prevalent in the adult rare disease population (3.4%) than in children (1.2%).

Whilst cross referencing between ORPHAcodes and ICD-10 in health administrative datasets facilitated international comparison and provided an overview of rare disease burden, it did not allow for in-depth analysis. The estimation of 1.5% of the Hong Kong population is noticeably lower than the reported estimates of 2.0% in the Western Australia study [11], and 6–8% from the European Commission estimates [2]. Since the CDARS system is not linked to the Hong Kong census data, results from this study may be an underestimate of the number of people living with rare diseases and should be considered as minimum values. Patients who have never used any services under the HA system, or those who have used other services such as outpatient, accident and emergency, and allied health services without the use of inpatient services were not included in this study. Total inpatient cost in this study was also likely underestimated owing to the unavailability of disease-specific inpatient cost listing in Hong Kong, and was calculated instead using the average inpatient cost within the HA. This included the clinical, biochemical and pathology investigation and general nursing fees only, and did not include other important costs such as pharmaceutical, inpatient allied health services and equipment costs. Moreover, the ORPHAcodes used in this study were only matched to 467 of the estimated 6000 to 8000 rare diseases [11], which further contributes to the underestimation of the true prevalence of rare disease. The overall rare disease prevalence remains unknown. Total healthcare cost attributed to rare disease is expected to be significantly higher with the inclusion of other healthcare services. In addition, disease-specific inpatient cost data is not available in the local setting, and that medication cost has not yet been taken into account in the current calculations, thus the true disparity between prevalence and proportion of healthcare cost is likely much higher.

## Conclusion

Despite its limitations, the current study adds to available literature by demonstrating the feasibility of adopting ORPHAcodes in the estimation of rare disease burden in Hong Kong. It also demonstrates the comparability of inpatient rare disease burden across different medical systems and helps to delineate rare disease distribution across ages. Subgroup analyses between the paediatric and adult population further provides important information for service planning in Hong Kong, such as the opening of Hong Kong Children Hospital in 2018.

Future areas for research include the extension of rare disease related healthcare cost calculation to intensive care, outpatient and medication related costs. The societal impact should also be considered by incorporating carer cost, production cost and trade-offs etc. However, costs and trade-offs associated with children is hard to measure, thus future research should also involve the development of instruments to capture these aspects. As public awareness for rare disease patients increases, it will be important to delineate the overlap between terms such as rare disease, children with medical complexities, children requiring palliative care, which are sometimes mistakenly or intentionally used interchangeably. As medical care for rare diseases advances and the children with once incurable conditions now live into and well beyond adulthood, further studies on transitional care will also be important.

## Abbreviations

CDARS: Clinical Data Analysis and Reporting System
HA: Hospital Authority
HKCTT: Hong Kong Clinical Terminology Table
ICD-10: the 10th version of the International Classification of Disease and Related Health Problems
ICD-10-AM: Australian modification of the 10th version of the International Classification of Disease and Related Health Problems
OMIM: Online Mendelian Inheritance in Man
ORDO: Orphanet Rare Disease Ontology
